# Targeting the Purinergic Axis with Phenolic Compounds to Disrupt the Oxidative-Inflammatory Cycle in Thyroid Cancer

**DOI:** 10.3390/ijms26178474

**Published:** 2025-08-31

**Authors:** Júlia Leão Batista Simões, Margarete Dulce Bagatini

**Affiliations:** 1 Graduate Program in Biochemistry, Federal University of Santa Catarina (UFSC), Florianópolis 88040-970, SC, Brazil; julialeaobatistasimoes@gmail.com; 2 Graduate Program in Medical Sciences, Federal University of Fronteira Sul, Chapecó 89815-899, SC, Brazil

**Keywords:** thyroid neoplasms, tumor microenvironment, purinergic signaling, phenolic compounds, drug resistance, immune checkpoint inhibitors

## Abstract

Thyroid cancer (TC), the most prevalent endocrine neoplasia, has shown a progressive incidence, highlighting the need for new therapeutic approaches—especially for radioiodine-refractory cases, often associated with mutations in genes such as *BRAF*, *RAS*, and *TP53*. This review proposes a mechanistic model that highlights two interrelated characteristics of the tumor microenvironment (TME): redox imbalance and chronic inflammation, key elements in tumor progression and treatment resistance. Thus, natural phenolic compounds, such as curcumin, quercetin, resveratrol, and epigallocatechin gallate (EGCG), function not as simple antioxidants but as pleiotropic agents that reprogram the TME. A central mechanism of action for these compounds is the modulation of the purinergic axis (CD39/CD73/adenosine), a critical immune-metabolic checkpoint. By selectively inducing lethal oxidative stress in tumor cells, suppressing pro-survival inflammatory pathways—such as that mediated by nuclear factor kappa B (NF-κB)—and destabilizing the immunosuppressive shield conferred by adenosine, certain phytochemicals demonstrate the potential to restore immune surveillance and promote tumor apoptosis. In this context, a critical analysis of the evidence related to targeting purinergic signals becomes essential, since pharmacological reinforcement of this pathway, especially when combined with immunotherapies based on immune checkpoint blockade, emerges as a promising strategy for overcoming therapeutic resistance.

## 1. Introduction

Thyroid cancer (TC) is the most prevalent endocrine neoplasia, with an incidence that has increased significantly in recent decades worldwide. This increase is predominantly attributed to papillary carcinoma (PTC), the most common histological subtype [1]. Although differentiated thyroid carcinomas (DTC), which include PTC and follicular carcinoma (FTC), generally have a favorable prognosis, a significant portion of cases progress to advanced, metastatic, or poorly differentiated forms, such as anaplastic carcinoma (APC). APC poses a considerable clinical challenge, with limited therapeutic options and high mortality rates [1].

Contemporary oncology research has established that oxidative stress (OS) and chronic inflammation are not mere epiphenomena but rather causal and promoting factors of malignant transformation and are considered “hallmarks of cancer” [2]. These two processes are intrinsically interconnected, forming a vicious cycle: OS, resulting from an imbalance between the production of reactive oxygen species (ROS) and the body’s antioxidant defenses, can initiate and sustain an inflammatory response [3]. The inflammatory microenvironment, characterized by high cytokine concentrations and immune cell infiltration, intensifies the production of reactive oxygen species (ROS), perpetuating a pathological cycle. This hostile environment favors cell proliferation, angiogenesis, drug resistance, and immune evasion and is regulated by complex signaling networks, among which the purinergic system stands out as a key element.

In this scenario, purinergic signaling, mediated by nucleotides such as adenosine triphosphate (ATP) and its degradation product, adenosine (Ado), functions as a critical sensor of TME conditions [4]. Under conditions of stress or tissue damage, ATP is released into the extracellular space, acting as a proinflammatory “danger” signal (DAMP). However, its subsequent hydrolysis to Ado by the ectonucleotidases CD39 (E-NTPDase1) and CD73 (Ecto-5′-nucleotidase) generates a potent immunosuppressive signal [5]. Given this dual function, modulation of the purinergic axis has become a promising therapeutic strategy. The purinergic system is not just an additional pathway but a central integrator that connects and amplifies the oxidative-inflammatory cycle in the TME. This interconnection suggests that modulating the purinergic axis may have a more significant therapeutic impact, as it aims to interrupt the entire pathological cycle, not just isolated components [4,5].

In this context, natural phenolic compounds, such as curcumin and resveratrol, go beyond antioxidant activity but rather serve as pleiotropic agents capable of reprogramming this axis and reversing immunosuppression. Redefining phenolic compounds as pleiotropic agents that reprogram the TME positions them as sophisticated therapeutics capable of altering fundamental biological characteristics of the tumor microenvironment [6]. This “reprogramming” capacity is crucial for combating cancer’s adaptive resistance mechanisms, giving them the potential to act as “smart drugs” that normalize the TME. Thus, by dissecting the multifaceted interaction between oxidative stress, inflammation, and purinergic signaling in the pathogenesis of thyroid cancer, this review will unify the therapeutic potential of these compounds in oncogenesis. A critical analysis of the literature will be conducted to substantiate the role of the CD39/CD73/adenosine axis and the P2X7 receptor as central therapeutic targets. We will explore, in particular, the translational potential of phenolic compounds as pharmacological modulators of these pathways. Finally, we will discuss preclinical evidence, their combined action with immunotherapies, and the need for nanoformulations to enable their clinical application, consolidating these phytochemicals as a new therapeutic frontline for thyroid cancer.

While a substantial body of research has independently explored the roles of oxidative stress, inflammation, and purinergic signaling in cancer, and numerous reviews have detailed the antineoplastic properties of phenolic compounds, a comprehensive framework connecting these elements within the specific context of thyroid cancer has been lacking. Previous work has often focused on singular aspects, such as the role of specific mutations or the antioxidant capacity of phytochemicals. This review, however, proposes a unique conceptual model that integrates these disparate fields. We uniquely posit that the purinergic axis is not merely another pathway but the central immunometabolic checkpoint through which the vicious cycle of oxidative stress and inflammation is maintained and amplified in the thyroid tumor microenvironment. Consequently, we redefine phenolic compounds not as simple antioxidants, but as sophisticated pleiotropic agents capable of reprogramming this entire axis, thereby offering a more holistic and mechanistically unified strategy to overcome therapeutic resistance.

## 2. Molecular Heterogeneity and Challenges in Thyroid Cancer

TC encompasses a spectrum of histological entities with distinct biological behaviors, originating predominantly from follicular cells [6]. Differentiated carcinomas, which include PTC and FTC, represent the vast majority of cases and generally have a favorable prognosis. However, a fraction of these tumors evolve into APC, an undifferentiated neoplasm characterized by extreme aggressiveness, therapeutic resistance, and high mortality, being one of the most aggressive and lethal tumors in humans [6]. The molecular pathogenesis of thyroid cancer follows a multistep progression model, initiated by mutations in key signaling pathways [6]. Activation of the MAP kinase (MAPK) pathway, through the BRAF V600E mutation or chromosomal rearrangements such as RET/PTC, is a canonical event in PTC [6,7,8]. Mutations in the *RAS* gene or the PAX8/PPARY fusion are more prevalent in follicular-pattern neoplasms. The transition to the anaplastic phenotype is often marked by the acquisition of an inactivating mutation in the *p53* tumor suppressor gene, overlapping with one of the initiating mutations [6]. Additionally, epigenetic alterations, such as methylation of tumor suppressor gene promoters, significantly contribute to dedifferentiation and progression. Even for mutations considered “actionable,” such as *BRAF V600E*, resistance mechanisms can emerge [7].

**Figure 1:**
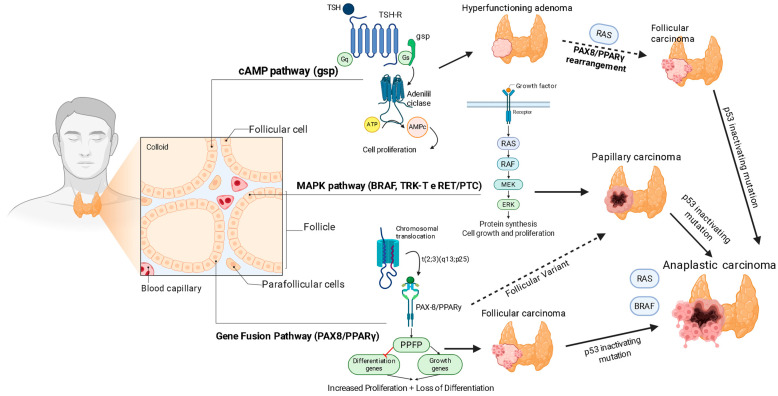
Genetic and signaling pathways in thyroid carcinogenesis. The left panel illustrates the normal thyroid gland anatomy, including follicular cells arranged in follicles surrounding colloid, parafollicular cells, and associated blood capillaries. Within the follicular cell, key signaling pathways are depicted. The cyclic AMP (cAMP) pathway, activated by thyroid-stimulating hormone (TSH) binding to its receptor (TSH-R) and subsequent G-protein (Gsp) activation of adenylyl cyclase, promotes cell proliferation. The mitogen-activated protein kinase (MAPK) pathway is activated by growth factors binding to receptor tyrosine kinases (e.g., rearranged during transfection/papillary thyroid carcinoma [RET/PTC] fusions, B-Raf proto-oncogene [*BRAF*]). It is crucial for protein synthesis, cell growth, and proliferation. The *PAX8/PPARγ* gene fusion is shown as a chromosomal translocation t(2;3)(q13;p25) leading to the expression of the *PAX8/PPARγ* fusion protein (PPFP), which increases proliferation and causes a loss of differentiation by impacting differentiation and growth genes. The right panel illustrates the progression of thyroid carcinomas driven by specific genetic alterations, including RAS proto-oncogene (*RAS*) and *BRAF* mutations, culminating in aggressive anaplastic carcinoma often characterized by an additional *p53* inactivating mutation. Hyperfunctioning adenomas and follicular carcinomas can arise from *RAS* mutations or *PAX8/PPARγ* rearrangements. Papillary carcinoma is frequently associated with RAS, BRAF, or RET/PTC mutations, with a follicular variant of papillary carcinoma also linked to the *PAX8/PPARγ* fusion. Notably, the development of aggressive anaplastic carcinoma is often characterized by the acquisition of a p53 inactivating mutation, in addition to pre-existing RAS or BRAF mutations, highlighting a crucial step in dedifferentiation and malignancy. Created with BioRender.com (accessed on 1 August 2025).

The complexity of the disease is evident in its molecular basis. A central clinical consequence of this dedifferentiation process is the loss of expression of the sodium/iodine symporter (NIS), the protein responsible for iodine uptake [6,9,10]. This event makes tumors refractory to radioactive iodine (RAI) therapy, the primary treatment modality for metastatic disease, creating a significant therapeutic vacuum [6,9,10]. The need for new therapeutic strategies for these aggressive forms is therefore pressing [6]. The limitation of current therapies, including tyrosine kinase inhibitors (TKIs), which, although effective, often lead to the development of resistance for CAT- and RAI-refractory tumors, poses a substantial clinical challenge, driving the search for innovative approaches (Figure 1).

In this context, natural phenolic compounds, such as curcumin, resveratrol, quercetin, and epigallocatechin-3-gallate (EGCG), have been widely studied for their antineoplastic properties [6]. Their efficacy stems from their pleiotropic action, with the ability to interfere with various cellular pathways, including the induction of apoptosis, the inhibition of angiogenesis, and the regulation of oncogenic pathways [6,11,12,13]. This versatility makes them promising candidates for targeting different stages of tumor progression, which is particularly relevant given the molecular heterogeneity characteristic of cancer.

The growing understanding that these compounds can “reprogram” the TME reinforces their therapeutic potential, suggesting a more comprehensive approach than simply neutralizing free radicals. The role of phenolic compounds goes beyond controlling general characteristics of the TME, such as oxidative stress and inflammation [12,13], reaching specific targets, such as restoring the expression of the iodine transporter (NIS). This indicates that these molecules may represent a complementary therapeutic strategy for addressing resistance related to specific mutations [10,12], either by directly modulating these pathways or by reversing the dedifferentiated state of tumor cells.

This approach positions them as agents capable of filling therapeutic gaps in the face of mutations considered “intractable” or acquired resistance to treatment [6]. The discussion delves into the mechanisms involved, with an emphasis on two interconnected and central characteristics of the TME: oxidative stress and inflammation. Furthermore, modulation of the purinergic axis, an immunometabolic signaling pathway, is proposed as a unifying and crucial mechanism to explain the therapeutic potential of these compounds in thyroid cancer [6].

## 3. The Oxidative Stress-Inflammation Axis: The Central Vulnerability of Thyroid Cancer

The TC microenvironment is defined by a profound homeostatic imbalance, in which oxidative stress (OS) and chronic inflammation form a self-sustaining axis that drives malignancy [14,15]. The very physiology of the thyroid gland makes it uniquely susceptible to this process. Thyroid hormone synthesis depends on the generation of hydrogen peroxide (H_2_O_2_), a potent reactive oxygen species (ROS), for the proper function of thyroid peroxidase (TPO). Consequently, follicular cells operate in a basal state of high oxidative flux, demanding robust antioxidant defenses to prevent cellular damage [14].

In TC cells, this condition is exacerbated by metabolic reprogramming, including the Warburg effect, which further elevates ROS production [6]. To survive, tumor cells overexpress defense systems such as the glutathione (GSH) pathway. However, this balance is precarious [15]. Clinical studies demonstrate that patients with TC have a reduced total antioxidant status (TAS) and an increased total oxidant status (TOS), making the oxidative stress index (OSI) a robust biomarker for the disease, stronger than in autoimmune conditions such as Hashimoto’s thyroiditis [15].

Once a pathological OS is established, it drives carcinogenesis through multiple mechanisms. ROS, often generated by overexpressed enzymes such as NADPH oxidase 4 (NOX4) and the Dual Oxidase (DUOX) family, cause direct damage to macromolecules [16]. In DNA, this leads to the formation of lesions such as 8-hydroxy-2′-deoxyguanosine (8-OHdG), a biomarker of genomic instability and carcinogenic risk in TC [17].

In addition to genetic damage, ROS function as signaling molecules that activate central oncogenic pathways, such as MAPK and PI3K/AKT. They do this, in part, by oxidatively inactivating protein tyrosine phosphatases (PTPs), which normally suppress these pathways [16]. Factors such as estrogen can exacerbate this scenario, with estrogen receptor alpha (ERα) activating the ROS and ERK1/2 pathways to inhibit apoptosis in PTC cells [17]. This sets up a vicious cycle: oncogenic pathways boost metabolism and ROS production, which then strengthen oncogenic signaling.

The oxidative environment created by this process inevitably draws in and activates immune cells, leading to chronic inflammation that further drives tumor progression [15,16,17,18]. The Nuclear Factor kappa B (NF-κB) pathway is the central link connecting inflammation to cancer survival. In CT, especially in advanced and anaplastic forms, NF-κB is constitutively active, both via the canonical (cytokine-activated) and non-canonical (lymphotoxin-β) pathways, providing a potent anti-apoptotic signal and promoting the self-renewal of tumor stem cells [18,19].

The inflammatory microenvironment, sustained by mutations such as BRAF V600E, is rich in cytokines such as interleukin-6 (IL-6) and CXCL8 [15,16]. These molecules not only promote proliferation but also induce tumor cell dedifferentiation. By suppressing the expression of specific thyroid genes (such as the sodium iodide symporter—NIS), inflammation directly causes resistance to radioactive iodine (RAI) therapy, one of the most significant clinical challenges in the treatment of advanced TC [16]. Understanding that chronic inflammation can directly cause RAI resistance by suppressing NIS expression is a critical point [10]. This means that any therapeutic strategy to overcome RAI resistance must address the underlying inflammatory microenvironment.

The dependence of TC cells on a high ROS state to survive and proliferate also represents their greatest vulnerability. They operate dangerously close to a toxic ROS threshold, where any further increase can overwhelm their defenses and induce cell death. It is in this “redox paradox” that the mechanism of action of many phenolic compounds resides [14,15,16]. The description that tumor cells operate in a “fragile equilibrium” of redox homeostasis, dangerously close to a toxic ROS threshold, reveals that the tumor’s very strength (high metabolic activity and ROS production) is also its weakness [17,18]. The therapeutic strategy, in this sense, is not to reduce oxidative stress but rather to push it beyond a critical threshold.

**Figure 2:**
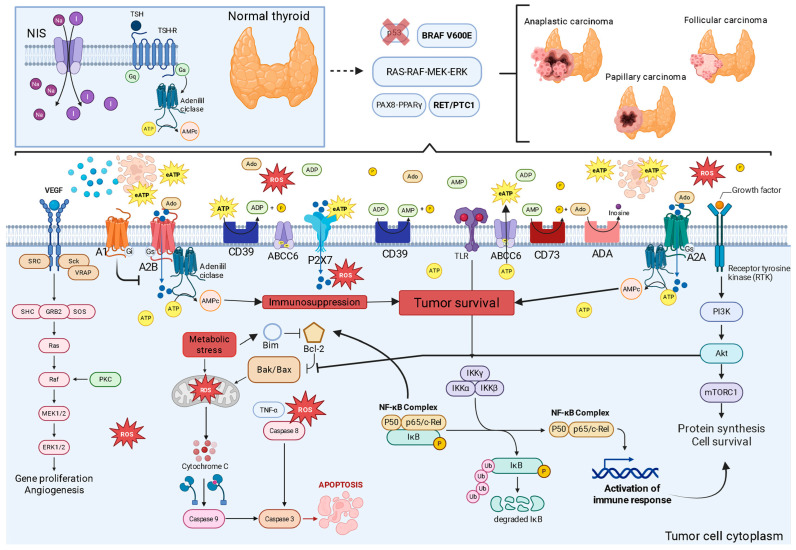
Molecular pathways involved in thyroid cancer progression, immune evasion, and potential therapeutic targets. The top left panel illustrates a normal thyroid cell, depicting the function of the Sodium-Iodide Symporter (NIS) for iodide uptake and the TSH/TSH-R/cAMP pathway. The top right panel indicates key genetic alterations frequently in anaplastic, follicular, and papillary carcinomas: BRAF V600E, RAS-BRAF-MEK-ERK pathway mutations, PAX8-PPARγ rearrangements, and RET/PTC fusions, which contribute to the development of anaplastic, follicular, and papillary carcinomas. The central diagram below details complex signaling networks within the tumor microenvironment (TME) that promote tumor survival and immunosuppression. Extracellular ATP (eATP), released due to metabolic stress or cellular damage, can activate purinergic receptors like P2X7 or stimulate adenosine production via CD39 and CD73. Adenosine (Ado) can bind to A2B or A2A adenosine receptors. A2B activation, often via VEGF stimulation, can lead to the production of eATP, contributing to a cycle of pro-tumorigenic signaling. A2A activation, stimulated by growth factors and RTKs, activates the PI3K/Akt/mTORC1 pathway, promoting protein synthesis and cell survival, and also contributes to immunosuppression. CD39 and CD73 facilitate the conversion of eATP to adenosine, a potent immunosuppressant. Intracellularly, metabolic stress and reactive oxygen species (ROS) can influence pro-apoptotic (e.g., Bak/Bax) and anti-apoptotic (e.g., Bcl-2) pathways. ROS can also contribute to the activation of the nuclear factor kappa B (NF-κB): p50/p65/c-Rel, which typically promotes an anti-apoptotic response and can also activate immune responses. However, persistent or dysregulated NF-κB can contribute to tumor survival. Ultimately, these pathways converge to promote tumor survival by fostering gene proliferation, angiogenesis, and immunosuppression, while inhibiting apoptosis. Created with BioRender.com (accessed on 1 August 2025).

Contrary to their popular classification as “antioxidants,” at pharmacological concentrations and in the context of the TME, these compounds can act as selective prooxidants. By catalyzing ROS production through Fenton-like reactions or inhibiting crucial antioxidant enzymes such as glutathione reductase, they push the tumor cell beyond its redox tipping point. This lethal oxidative stress induces irreparable damage and activates apoptosis via the mitochondrial pathway [6]. This selective action, which exploits an intrinsic feature of cancer biology, differentiates phenolic compounds from conventional therapies and offers a promising strategy to break the vicious cycle of oxidative stress and inflammation that fuels thyroid cancer (Figure 2).

### 3.1. Limitations of Current Evidence

While the link between the oxidative-inflammatory axis and thyroid cancer is compelling, it’s important to critically assess the supporting evidence.

#### 3.1.1. Correlation vs. Causation

Much of the human data are correlational, showing a strong association between oxidative stress markers and the presence of thyroid cancer [2]. It remains challenging to definitively prove in humans whether this profound redox imbalance is a primary cause of malignant transformation or a consequence of the established disease.

#### 3.1.2. Reliance on Preclinical Models

The detailed mechanisms, such as the specific roles of NOX4 and DUOX enzymes in generating ROS, have been elucidated primarily through in vitro and animal models [19]. These models, while valuable, may not fully replicate the complex interplay of diverse immune and stromal cells found in the human tumor microenvironment.

#### 3.1.3. The Challenge of Therapeutic Selectivity

The “redox paradox”—the strategy of pushing cancer cells past a toxic ROS threshold—is a powerful therapeutic hypothesis [2]. However, the key translational challenge is developing drugs that can selectively induce lethal oxidative stress in tumor cells without causing significant off-target damage to healthy tissues, a hurdle that has so far limited the clinical application of this approach.

## 4. Purinergic Signaling: The Immunometabolic Switch of Thyroid Cancer

In the intricate ecosystem of the TME, purinergic signaling functions as a critical communication system that thyroid cancer cells manipulate to orchestrate their survival and immune evasion [4]. This system, composed of ligands such as ATP and adenosine, their respective membrane receptors (P2 and P1), and a series of regulatory ectoenzymes, functions as a biological sensor of tissue status [4,5]. Within the thyroid TME, conditions of metabolic stress, hypoxia, chronic inflammation, and a high rate of cell death lead to the massive release of ATP into the extracellular space. This event transforms ATP from its function as an intracellular energy currency into a potent external signaling molecule. Initially, this extracellular ATP (eATP) acts as an alarm signal, a Damage-Associated Molecular Pattern (DAMP), alerting the immune system to imminent danger [4,5,20]. By activating P2 receptors, particularly P2X7, on immune cells, eATP triggers an acute proinflammatory response, a “red alert” designed to eliminate damaged or malignant cells [4,20].

However, the malignant ingenuity of cancer lies in its ability to co-opt the host’s control mechanisms. To prevent excessive tissue damage from uncontrolled inflammation, the purinergic system possesses an intrinsic “off switch,” and it is precisely this switch that the tumor exploits [21,22]. Tumor cells and allied stromal cells overexpress a cascade of ectonucleotidases on their surface, notably CD39 and CD73. These enzymes work in concert to disarm the ATP danger signal. CD39 initiates the process by hydrolyzing proinflammatory eATP into adenosine monophosphate (AMP) [22]. Subsequently, CD73, often the rate-limiting enzyme in the pathway, completes the conversion, generating adenosine (Ado) [23,24]. This process efficiently transforms a danger signal into a powerful immunosuppressive signal. The expression of these enzymes is dynamically amplified by TME conditions, such as hypoxia and cytokines like TGF-β, creating a feedback loop that floods the tumor with protective Ado [22,23].

The relevance of this axis to thyroid cancer is unequivocal: studies demonstrate that CD73 is significantly overexpressed in papillary carcinomas (PTC), especially at the invasive tumor fronts, and its presence is linked to a poorer clinical prognosis, validating it as a high-priority therapeutic target [22,24,25,26]. This locally produced Ado is the chief architect of immune paralysis in the thyroid TME. By binding to its high-affinity receptors, primarily A2A receptor (A2AR) and A2B receptor (A2BR), on the surface of crucial effector immune cells—such as cytotoxic T lymphocytes and Natural Killer (NK) cells—it triggers an intracellular signaling cascade via increased cyclic AMP (cAMP) [4,5]. This increase in cAMP acts as a potent inhibitory signal, paralyzing the proliferation, activation, and cytotoxic function of these cells, inducing a state of profound anergy or “exhaustion” [25]. To further aggravate this scenario, Ado also promotes the expansion and function of suppressor cell populations, such as regulatory T cells (Tregs), which, in turn, express high levels of CD39, perpetuating Ado production and deepening the state of immune paralysis [26].

Due to this ability to deactivate anti-tumor surveillance, the CD39/CD73/Adenosine axis is now considered a fundamental immuno-metabolic checkpoint, whose importance is analogous to that of the well-known PD-1/PD-L1 pathway [24,25,26]. The explicit comparison of the purinergic axis with the PD-1/PD-L1 pathway suggests that tumors have developed multiple, and possibly redundant, immune evasion mechanisms. If one checkpoint, such as PD-1/PD-L1, is blocked, the “adenosine shield” may continue to suppress the immune response [25,26]. This observation highlights the need for combined therapies, where monotherapy of a single checkpoint may be insufficient if other potent immunosuppressive pathways remain active.

However, the purinergic story is not just about immunosuppression. Initial ATP, prior to its degradation, activates the P2X7 receptor (P2X7R), which displays a paradoxical dual function that thyroid cancer cells manipulate for their benefit [27]. Stimulation of P2X7R with low concentrations of eATP results in the opening of an ion channel that promotes Ca^2+^ influx, activating survival and proliferation pathways such as PI3K/Akt and MAPK/ERK [27]. In contrast, sustained stimulation with high concentrations of eATP induces a drastic conformational change, leading to the formation of a large cytotoxic pore that causes the collapse of ionic gradients and cell death. Tumor cells resolve this paradox by expressing splicing variants (such as P2X7R) or non-functional forms that are deficient in death pore formation but retain the pro-growth signaling capacity [28].

Thus, the tumor transforms a potential host weapon into a tool for its expansion. In thyroid cancer, increased P2X7R expression is directly correlated with greater tumor aggressiveness: its activation is a canonical trigger for NLRP3 inflammasome assembly, releasing the cytokines IL-1β and IL-18; it stimulates ROS production via NADPH oxidase, fueling the oxidative stress cycle; and it promotes profound metabolic reprogramming, increasing the Warburg Effect [28]. In thyroid cancer, increased P2X7R expression and specific genetic polymorphisms are correlated with greater aggressiveness, confirming its role as a critical mediator of tumor progression.

The functional duality of P2X7R, which can be pro-survival at low ATP concentrations and cytotoxic at high concentrations, represents a complex therapeutic challenge and opportunity. The ability of cancer cells to manipulate this receptor, expressing variants that promote growth but avoid death, suggests that future strategies may require high specificity. Rather than simply blocking P2X7R, exploiting its cytotoxic function may require precise targeting approaches, perhaps through nanoformulations that deliver agonists only to the tumor microenvironment.

### 4.1. Therapeutic Implications: Targeting the Purinergic Axis

This detailed understanding of purinergic biology in cancer has spurred the development of immunotherapies with direct relevance for treating advanced thyroid cancer. The central strategy is to dismantle the “adenosine shield” to reactivate the anti-tumor immune response, a particularly crucial goal for immunologically “cold” or resistant thyroid tumors [26,29]. Clinical approaches include the use of monoclonal antibodies or small molecules to inhibit CD73 (e.g., Oleclumab), blocking the final step of Ado production; inhibition of CD39 (e.g., TTX-030), a “double-hit” tactic that not only prevents Ado generation but also preserves proinflammatory ATP; or the use of A2AR antagonists (e.g., Ciforadenant) to block the effects of Ado on immune cells directly [29]. The true therapeutic promise for thyroid cancer, however, may lie in rational combination with immune checkpoint inhibitors (ICIs).

The adenosine pathway is a fundamental mechanism of primary and acquired resistance to ICIs. The Phase II clinical trial COAST provided robust proof-of-concept for this combined action, demonstrating that the combination of an anti-CD73 with an anti-PD-L1 significantly improved outcomes in lung cancer patients [29,30,31]. Therapeutic modulation of P2X7R, in turn, remains a complex challenge; while antagonism to block its pro-growth effects has shown preclinical success, clinical results in other diseases have been disappointing [30,31]. The alternative and bolder strategy of using agonists to force the opening of the death pore, although theoretically powerful, carries significant risks of toxicity, highlighting the complexity and richness of therapeutic opportunities contained in modulating this central axis of the tumor microenvironment. A two-enzyme cascade on the cell surface orchestrates the adenosinergic axis. The process is initiated by the CD39, which hydrolyzes eATP and ADP to AMP—a crucial step in the pathway, controlling the flow of nucleotides for degradation.

Subsequently, the enzyme CD73 completes the cascade, hydrolyzing AMP to generate immunosuppressive Ado and inorganic phosphate [31]. Together, these two enzymes function in concert to efficiently convert a proinflammatory signal—ATP—into a profoundly anti-inflammatory and immunosuppressive signal—adenosine [4,24,31]. The expression of CD39 and CD73 is not static; prevalent conditions in the TME dynamically regulate it [31,32]. Factors such as hypoxia, oxidative stress, and inflammatory cytokines, notably transforming growth factor beta (TGF-β), are potent inducers of both enzyme expressions in tumor and stromal cells [31,33]. This regulation creates an adaptive mechanism by which the tumor responds to stress, amplifying its ability to generate protective adenosine.

In the specific context of thyroid cancer, the evidence is increasingly evident. Studies demonstrate that CD73 is significantly overexpressed in PTC tissues compared to normal adjacent thyroid tissue [31,33]. This overexpression is not merely a passive marker; it is functionally associated with a poorer clinical prognosis, including a higher rate of disease recurrence and the presence of lymph node metastases. At the cellular level, CD73 inhibition in PTC cell lines has been shown to block adenosine-induced proliferation and migration, confirming its direct role in promoting tumor aggressiveness [25,31,32,33].

The CD39/CD73 axis represents a sophisticated immune evasion mechanism whereby tumors co-opt the host’s “danger” signal—ATP and transform it into a “safety” signal—Ado. The generated Ado exerts its immunosuppressive effects primarily through binding to the A2AR, which is highly expressed on the surface of crucial effector immune cells, such as T cells and Natural Killer (NK) cells [16,34]. Activation of A2AR signaling has direct and devastating consequences for the anti-tumor response. It inhibits the proliferation, activation, and cytotoxic function of CD8+ T cells and NK cells, which are the immune system’s primary soldiers in the fight against cancer [34].

Simultaneously, Ado acts to strengthen the suppressive side of the immune response. It promotes the activity and expansion of immunosuppressive cell populations, such as regulatory T cells (Tregs) and myeloid-derived suppressor cells (MDSCs), which infiltrate the TME and actively suppress the anti-tumor response. Notably, Tregs express high levels of CD39 on their surface, establishing a positive feedback loop that continuously amplifies Ado production and deepens the state of immunosuppression [26,34]. Therefore, inhibiting this axis offers a “double-hit” strategy: it not only removes the protective “adenosine shield” but also restores the “danger” signal of ATP, reactivating immune surveillance [27].

Understanding this crucial role of ATP as a “danger” signal, it is important to consider its interaction with specific receptors. One such receptor, the P2X7 receptor (P2X7R), is an ATP-gated ion channel that exhibits remarkable functional duality, a “split personality” that depends on the concentration and duration of its ligand’s stimulation. This duality represents a “life or death” decision point that cancer cells learn to manipulate to ensure their survival [21]. Stimulation with low to moderate concentrations of eATP, or for short periods, results in the opening of a selective cationic channel, allowing the influx of Ca^2+^ and Na^+^ ions. This calcium influx acts as a second messenger, activating pro-survival and pro-proliferative intracellular signaling pathways, such as the PI3K/Akt and MAPK/ERK pathways [30].

In contrast, sustained stimulation with high concentrations of eATP, such as those found in the TME, induces a drastic conformational change in the receptor, leading to the formation of a large nonselective pore. The opening of this pore is catastrophic for the cell, causing the collapse of ionic gradients, osmotic stress, and ultimately, cell death by necrosis or apoptosis. The paradox is that many cancers, including thyroid cancer, not only survive but also thrive in an ATP-rich environment, often overexpressing P2X7R [30]. The solution to this paradox lies in the ability of tumor cells to functionally “edit” the receptor. They frequently express splicing variants (such as P2X7R) or non-functional forms (nfP2X7) that are deficient in death pore formation but retain the pro-growth ion channel signaling capacity. In this way, the tumor transforms a weapon pointed against itself into a tool for its proliferation [23,31].

P2X7R functions as a central hub that integrates purinergic signaling with inflammation and oxidative stress pathways. It is a canonical activator of the NLRP3 inflammasome, an intracellular multiprotein complex. P2X7R activation, through K^+^ efflux, triggers inflammasome assembly, caspase-1 activation, and the subsequent cleavage and release of the potent proinflammatory cytokines interleukin-1β (IL-1β) and interleukin-18 (IL-18), which play complex roles in promoting and suppressing tumorigenesis [25,26]. Furthermore, P2X7R activation directly stimulates ROS production, primarily through the activation of the NADPH oxidase enzyme complex, establishing a direct and mechanistic link between extracellular danger detection (eATP) and intracellular oxidative stress generation [22,23,27]. P2X7R also acts as a “metabolic sensor” that allows cancer cells to adapt their metabolism to TME conditions. Tonic stimulation of the receptor promotes profound metabolic reprogramming, simultaneously increasing the rate of aerobic glycolysis (Warburg Effect) and mitochondrial oxidative phosphorylation (OXPHOS) [27]. This metabolic flexibility confers a significant growth and survival advantage to tumor cells, especially in environments with fluctuations in nutrients and oxygen [28].

**Figure 3:**
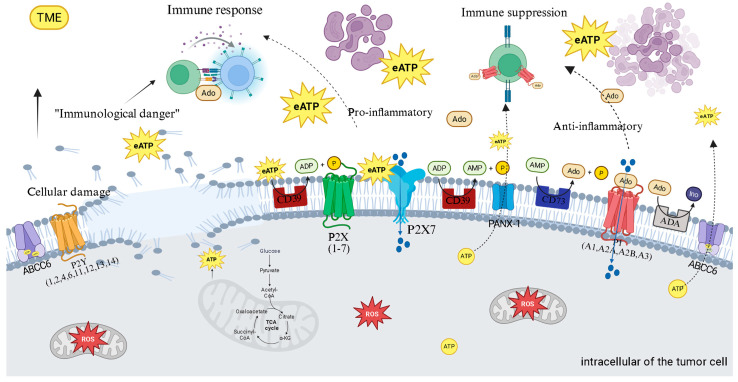
Regulation of extracellular ATP (eATP) metabolism and its impact on the tumor microenvironment (TME). The top panel illustrates the enzymatic cascade involved in eATP degradation. eATP is sequentially hydrolyzed by ectonucleotidases, primarily CD39 (E-NTPDase1), to ADP, then to AMP. Subsequently, CD73 (ecto-5′-nucleotidase) converts AMP to adenosine (Ado). Ado can then be further metabolized to inosine by adenosine deaminase (ADA). The bottom panel details the complex roles of eATP within the TME, highlighting its dual functions in immune activation and suppression. Cellular damage or stress can lead to the release of intracellular ATP into the extracellular space via channels like ABCB6, Pannexin-1 (PANX-1), or other mechanisms, creating an ‘immunological danger’ signal. High concentrations of eATP can activate various purinergic receptors on immune cells, including P2X (ligand-gated ion channels, e.g., P2X1–7) and P2Y (G-protein coupled receptors, e.g., P2Y1,2,4,6,11,12,13,14), leading to proinflammatory responses and the generation of reactive oxygen species (ROS). Conversely, the rapid hydrolysis of eATP by CD39 and CD73 generates adenosine, which can activate adenosine receptors (A1, A2A, A2B, A3) on immune cells, leading to immune suppression and an anti-inflammatory microenvironment. This intricate balance of eATP and Ado signaling dictates the immune response within the TME. Created with BioRender.com (accessed on 1 August 2025).

The relevance of P2X7R to TC is well-documented. Studies demonstrate that P2X7R expression is significantly increased in PTC compared to adjacent normal tissue. Furthermore, specific polymorphisms in the *P2X7R* gene have been correlated with greater disease aggressiveness. From a functional perspective, P2X7R activation in TC cells has been shown to directly stimulate the oncogenic MAPK/ERK pathway and increase the expression of cyclin D1, a key cell cycle regulator, thereby directly promoting tumor proliferation. These data position P2X7R as an important mediator of TC progression and a potentially valuable therapeutic target [20,23] (Figure 3). The true therapeutic promise for thyroid cancer, however, may lie in combining these strategies with existing treatments. Furthermore, this strategy is gaining traction in other endocrine malignancies, such as neuroendocrine neoplasms, where the adenosinergic pathway has also been validated as a novel therapeutic target [35].

Intriguingly, the therapeutic strategy of dismantling the “adenosine shield” with synthetic inhibitors is mirrored by the natural action of several phenolic compounds. These phytochemicals are not blunt instruments but act as sophisticated modulators of this specific immune checkpoint [6]. For instance, quercetin and EGCG have been identified as direct inhibitors of CD73 [36,37], the final enzyme responsible for generating adenosine [23]. Curcumin takes a broader approach, decreasing the expression of both CD39 and CD73 [37]. The downstream immune consequence of this targeted action is profound: by blocking adenosine production, these compounds prevent the paralysis of cytotoxic T lymphocytes and NK cells [5], restoring the immune system’s ability to recognize and eliminate tumor cells [31]. This positions phenolics as natural immunotherapeutic agents that reprogram the tumor microenvironment by directly manipulating the purinergic axis [12].

### 4.2. Therapeutic Strategies Based on Purinergic Modulation

The escalating understanding of the central role of purinergic signaling in immunosuppression and cancer progression has propelled the development of a novel class of therapeutic agents. The clinical strategy is rapidly evolving, moving away from monotherapy and embracing rational combinations that recognize the purinergic pathway as a critical modulator of the TME rather than an autonomous tumor driver. Among these, three primary approaches are being explored to dismantle the immunosuppressive adenosine pathway [31].

The utilization of CD73 inhibitors represents a more advanced strategy in clinical development, as it includes monoclonal antibodies, such as Oleclumab (MEDI9447; sourced from AstraZeneca, developed by US-Based MedImmune), and small molecule inhibitors. The objective is to block the final step of Ado production, thereby reducing its concentration in the TME and alleviating T-cell and NK-cell suppression [32]. Furthermore, CD39 inhibitors, which act at the initial and rate-limiting step of the cascade, offer a desirable strategy. Inhibitors like TTX-030 and IPH5201 provide a dual benefit: they not only prevent the generation of immunosuppressive Ado but also increase the concentration of proinflammatory ATP, which can potentiate the anti-tumor immune response [33].

A2AR antagonists represent an approach that targets the final step of the pathway, directly blocking the effects of Ado on its target cells. Agents such as Ciforadenant and the dual A2A/A2B antagonist Etrumadenant are designed to “release the brakes” on immune cells, restoring their ability to attack the tumor, even in the presence of Ado [32,33,34].

### 4.3. Rational Combinations with Immune Checkpoint Inhibitors

Initial clinical trial data have demonstrated that monotherapy with agents targeting the adenosine pathway generally yields modest efficacy. The true therapeutic promise lies in their combination with other treatment modalities, especially with ICIs, such as anti-PD-1 and anti-PD-L1 antibodies. The adenosine pathway is now recognized as a fundamental mechanism of both primary and acquired resistance to ICIs [31,37]. An adenosine-flooded TME can maintain T cells in a suppressed state, even when the PD-1 brake is released [34].

The rationale for combination therapy is therefore synergistic, and this has been demonstrated with strong clinical evidence in other cancers, which provides a compelling, albeit indirect, blueprint for thyroid cancer. The Phase II COAST clinical trial provided robust proof-of-concept for this strategy. In this study, the combination of oleclumab (anti-CD73) with durvalumab (anti-PD-L1) demonstrated a significant improvement in progression-free survival [29]. However, a critical gap remains, as similar large-scale clinical trials dedicated to thyroid cancer are yet to be conducted. While the biological principle is sound, the specific efficacy and patient selection criteria for this combination in BRAF-mutated PTC versus anaplastic thyroid carcinoma, for instance, are unknown. The success in lung cancer serves as a strong translational hypothesis, but direct clinical validation in thyroid cancer is a crucial and currently missing step. The understanding that the adenosine pathway is a mechanism of primary and acquired resistance to ICIs emphasizes the need for dynamic therapeutic strategies. Inhibition of the purinergic axis can act as a “sensitizer” for ICIs, improving initial response rates and offering a crucial line of attack to resensitize tumors that have developed resistance over time.

Therapeutic modulation of P2X7R presents a unique challenge due to its biphasic nature [28,30]. The most intuitive approach involves using antagonists to block the trophic and pro-proliferative effects mediated by ion channel activation. Several antagonists have shown efficacy in reducing tumor growth in preclinical models. However, clinical trials with P2X7R antagonists for inflammatory diseases have generally not achieved the expected results, and a recent 2025 update on the clinical landscape highlights the ongoing challenges in translating P2X7R antagonism into effective cancer therapy [23,30]. A more audacious and innovative alternative strategy would be to use potent agonists or positive allosteric modulators that could force the opening of the cytotoxic pore of P2X7R, selectively inducing cancer cell death. While theoretically powerful, this approach carries significant risks of systemic toxicity. It requires a much deeper understanding of the receptor’s biology and its expression variants in the specific context of each tumor [31] (Table 1).

Furthermore, the clinical translation of purinergic inhibitors is maturing beyond initial efficacy studies to address more complex challenges. Current research is intensely focused on identifying predictive biomarkers to select patients most likely to benefit and on understanding the nuanced roles of targets like CD39, which can have both pro- and anti-tumorigenic functions depending on the context [32,33,34]. This highlights a necessary shift from broad application to a more precise, biomarker-driven strategy to overcome the primary and acquired resistance that limits the success of immunotherapy in solid tumors [35].

### 4.4. Limitations of Current Evidence

The rationale for targeting the purinergic axis is strong, but the evidence base in thyroid cancer specifically has significant limitations.

#### 4.4.1. Lack of Thyroid-Specific Clinical Data

This is the most critical gap. The promising clinical data for purinergic inhibitors, such as the COAST trial, comes from non-small cell lung cancer, not thyroid cancer [29]. The entire therapeutic strategy for thyroid cancer is an extrapolation based on preclinical data; there are currently no dedicated clinical trials of CD73/CD39 inhibitors in this patient population.

#### 4.4.2. Tumor Heterogeneity Is Understudied

While CD73 is known to be overexpressed in papillary thyroid carcinoma [22], the expression patterns and functional importance of the complete purinergic machinery across different histological and molecular subtypes remain poorly characterized.

#### 4.4.3. Potential for Therapeutic Resistance

The manuscript acknowledges that adenosine can be produced through alternative pathways, such as via CD38. This points to a potential mechanism of acquired resistance where tumors could bypass a CD39/CD73 blockade, a possibility that requires further investigation in thyroid cancer models [31].

## 5. Phenolic Compounds as Pleiotropic Modulators in Thyroid Cancer

Initial clinical trial data have demonstrated that monotherapy with agents targeting the adenosine pathway generally yields modest efficacy [34]. The true therapeutic promise lies in their combination with other treatment modalities, especially with ICIs, such as anti-PD-1 and anti-PD-L1 antibodies. The adenosine pathway is now recognized as a fundamental mechanism of both primary and acquired resistance to ICIs [31].

The rationale for combination therapy is therefore synergistic, and this has been demonstrated with strong clinical evidence in other cancers, which provides a compelling, albeit indirect, blueprint for thyroid cancer. The Phase II COAST clinical trial provided robust proof-of-concept for this strategy in non-small cell lung cancer, where the combination of oleclumab (anti-CD73) with durvalumab (anti-PD-L1) demonstrated a significant improvement in progression-free survival [29]. However, a critical gap remains, as similar large-scale clinical trials dedicated to thyroid cancer are yet to be conducted. While the biological principle is sound, the specific efficacy and patient selection criteria for this combination in BRAF-mutated PTC versus anaplastic thyroid carcinoma, for instance, are unknown. The success in lung cancer serves as a strong translational hypothesis, but direct clinical validation in thyroid cancer is a crucial and currently missing step [12].

### 5.1. Compounds Phenolics

#### 5.1.1. Curcumin

Curcumin, a polyphenol, acts as a potent inhibitor of master survival pathways, such as the PI3K/Akt/mTOR pathway, which is crucial for proliferation and resistance to apoptosis, especially in aggressive tumors like APC [6,38]. Furthermore, curcumin potently inhibits the activation of the transcription factor NF-κB, a key regulator of inflammation and cell survival [6,19]. Innovatively, recent studies indicate that curcumin also directly impacts the purinergic axis by decreasing the expression of CD39, CD73, and the A2AR, thereby cutting off the production and signaling of immunosuppressive Ado [6,37,38,39]. The anticancer potential of curcumin has been extensively demonstrated in the laboratory, as in vitro studies using PTC cell lines demonstrated effective suppression of proliferation, migration, and invasion [38,40]. Furthermore, in vivo murine models show that curcumin enhances antitumor immunity in anaplastic carcinoma xenografts, demonstrating synergy with anti-PD-1 therapy [37]. However, it is crucial to recognize that these findings are preclinical. A significant translational gap for curcumin, as for other phenolics, is its low systemic bioavailability, which poses a major challenge to achieving the therapeutic concentrations used in these laboratory studies in human patients [41]. Although its mechanisms are compelling, robust clinical data, specifically in thyroid cancer patients, are still lacking, highlighting a critical area for future research [42].

Regarding oxidative stress, treatment of tumor cells with curcumin induces high levels of ROS production and accumulation, leading to redox imbalance and the death of malignant cells [6,17,38]. As a result, it can cause mitochondrial damage and trigger various types of cell death, such as apoptosis, autophagy, ferroptosis, and pyroptosis [17]. Specifically concerning thyroid cancer, curcumin notably suppresses proliferation, migration, and invasion in papillary thyroid carcinoma (PTC) cells [39,43]. It induces autophagic cell death in human thyroid cancer cells through the activation of MAPK and inhibition of mTOR pathways [38]. Additionally, curcumin enhances anti-tumor immunity in APC by elevating CD8+ T cell function and downregulating the AKT/mTORC1/STAT3/PD-L1 axis [37,44]. It also inhibits metastasis in human papillary thyroid carcinoma cells through the downregulation of the TGF-β/Smad2/3 signaling pathway [38].

#### 5.1.2. Resveratrol

Resveratrol is known for its ability to activate SIRT1, a nuclear enzyme that regulates metabolism and epigenetics, and can induce cell redifferentiation [6,45]. Its most direct action in the context of this work is the modulation of adenosine receptors [6,46]. Resveratrol promotes an increase in the levels of the inhibitory A1 receptor and a reduction in the stimulatory A2AR, rebalancing adenosine signaling toward an anti-tumor profile [6,47]. Additionally, it induces cell death through oxidative stress and caspase activation [6,48].

In the context of thyroid cancer, resveratrol has demonstrated a promising effect in increasing ^131^I-induced cell death in thyroid cancer cells, while exhibiting a protective effect on normal cells against ^131^I toxicity [48]. It induces the expression of differentiation markers (TTF1, TTF2, Pax8, and sodium/iodide symporter (NIS)) in anaplastic thyroid carcinoma through the activation of Notch1 signaling, thereby suppressing cell growth [49]. Resveratrol has also been reported to reverse multidrug resistance in cancer cells [47].

Similarly, the evidence for resveratrol is confined to preclinical models. Its ability to induce redifferentiation markers, including the NIS, in anaplastic thyroid carcinoma cell lines is a key finding, as it suggests a strategy to resensitize tumors to radioactive iodine [50]. While this is a highly significant potential application, this effect has only been observed in vitro. A major caveat to these promising findings is resveratrol’s notoriously poor bioavailability [41,51]. This limitation was clearly demonstrated in an animal study where free resveratrol failed to inhibit tumor growth, whereas a nanoparticle formulation succeeded [51,52]. This directly illustrates that the therapeutic concept is viable but wholly dependent on overcoming the pharmacokinetic hurdle. Without effective delivery systems, the translational potential of resveratrol remains limited.

#### 5.1.3. Quercetin

Quercetin, a flavonoid, demonstrates an exquisite attack strategy against the purinergic system. It inhibits the activity of CD73, the final enzyme in Ado production [6,48]. Further upstream, evidence suggests that quercetin may limit ATP efflux from the tumor cell, possibly by inhibiting ABC family transporters [6,49]. This dual action, combined with its ability to induce apoptosis via NAG-1/GDF15, makes it a promising agent [6,53,54].

In terms of oxidative stress and inflammation, quercetin possesses potent antioxidant and anti-inflammatory properties [55,56]. It mitigates oxidative stress by neutralizing ROS [56]. It also downregulates NF-κB activity and modulates JAK/STAT signaling, increasing immune recognition of cancer cells and decreasing inflammation in the tumor microenvironment [56,57]. Specifically in thyroid cancer, quercetin induces anticancer activity by upregulating pro-NAG-1/GDF15 in differentiated thyroid cancer cells, leading to the induction of apoptosis and cell cycle arrest [54,58]. It can inhibit thyroid cancer cell proliferation and matrix metalloproteinase-3 (MMP3) expression under high glucose conditions [2]. It has also demonstrated strong anti-tumor efficacy in PTC cell lines [57].

#### 5.1.4. Epigallocatechin-3-Gallate (EGCG)

EGCG, the primary catechin in green tea, also acts on multiple targets. It can directly modulate the apoptosis machinery by altering the balance of Bcl-2 family proteins. It has also been shown to inhibit CD73 [6,59]. Its known ability to modulate T-cell differentiation can synergize with Ado reduction to restore immune function in the TME [6,59].

Regarding oxidative stress and inflammation, EGCG exhibits a dual function of potential antioxidant and prooxidant, with ROS generation considered essential for inducing apoptosis and inhibiting cancer cell growth [60,61,62]. It is also associated with the inhibition of nuclear factor-κB (NF-κB) signaling [59]. In the context of thyroid cancer, EGCG inhibits the growth and increases apoptosis of human thyroid carcinoma cells through the suppression of the EGFR/RAS/RAF/MEK/ERK signaling pathway [63]. It decreases the migration and invasion of human thyroid carcinoma cells [61,63]. EGCG can also induce epigenetic modifications by inhibiting DNA methyltransferase activity [59]. It inhibits proliferation and motility with concomitant loss of epithelial-mesenchymal transition markers [56]. CD73 expression is a characteristic of papillary carcinomas [61,63].

The ability of these phenolic compounds to influence a wide range of oncogenic pathways and biological processes in thyroid cancer, including proliferation, apoptosis, differentiation, metastasis, inflammation, oxidative stress, and immune evasion, positions them as sophisticated pharmacological agents [61,62]. The pleiotropic nature of phenolic compounds, which allows them to target multiple cancer pathways and hallmarks simultaneously, is particularly well-suited to combating the inherent heterogeneity and adaptive resistance mechanisms of thyroid cancer [64,65,66,67]. Unlike targeted therapies that compensatory pathways can circumvent, phenolics offer a “multi-pronged attack” approach that makes evasion by cancer cells more difficult, potentially leading to more durable responses [68].

A fundamental understanding that emerges is the direct modulation of the immunosuppressive purinergic axis by these compounds. Curcumin, by decreasing the expression of CD39, CD73, and A2A [6,39], resveratrol, by modulating A1 and A2A receptors [6,47,48,49], quercetin, by inhibiting CD73 activity and ATP efflux [6,48,49], and EGCG, by inhibiting CD73 [6,59], all demonstrate a specific and crucial molecular intervention in an immune checkpoint pathway. This specific molecular action, which disarms the “adenosine shield” used by tumors to evade immune surveillance, is a cornerstone for the immune reprogramming of the tumor microenvironment.

Furthermore, the action of phenolics as re-differentiating agents for RAI-refractory thyroid cancer represents a significant advance [65,67]. Resveratrol, for instance, activates SIRT1 and induces cellular redifferentiation [6,47], and, more specifically, induces the functional expression of the Notch1 protein and upregulates thyroid-specific genes, including NIS, in APC cells [49]. The ability of agents like resveratrol to induce cellular redifferentiation and restore NIS expression in RAI-refractory tumors represents a notable breakthrough [66,67]. Thus, phenolic compounds are understood not only as stand-alone therapies or sensitizers for immunotherapies but also as agents capable of rescuing or redirecting existing treatments that have lost efficacy due to tumor dedifferentiation, generating significant clinical implications for patients with advanced and previously untreatable thyroid cancer (Table 2).

A comprehensive understanding of the multi-target action of phenolic compounds, particularly their modulation of the purinergic axis, reveals highly impactful therapeutic avenues for thyroid cancer.

### 5.2. Summary of Mechanistic Focus

While a direct comparison of potency is not possible from the available data, the literature described in this review highlights a distinct mechanistic focus for each phenolic compound. This provides a qualitative way to compare their strategic applications. Curcumin is presented as the most comprehensive modulator of the purinergic production pathway, as it’s uniquely described as decreasing the expression of both CD39 and CD73 [39]. It’s also emphasized as a potent inhibitor of the master survival pathway NF-κB [18]. Resveratrol’s most distinct therapeutic angle is its function as a redifferentiating agent [46]. Its ability to restore NIS expression via Notch1 activation is a unique mechanism not attributed to the other compounds, positioning it as a prime candidate for strategies aimed at resensitizing tumors to RAI therapy [54,58].

Quercetin and EGCG are both highlighted as direct inhibitors of CD73 activity [38,40]. While their relative potencies are not specified, their action is focused on blocking the final, crucial step of immunosuppressive adenosine production. Quercetin is also noted for its potent general antioxidant properties [56] and its ability to potentially limit ATP efflux from the tumor cell [53]. This summary shows that while there’s overlap, each compound offers a primary strategic advantage based on the mechanisms emphasized in this review—from broad pathway suppression (Curcumin) to targeted redifferentiation (Resveratrol) to direct enzymatic inhibition (Quercetin and EGCG).

### 5.3. Resensitization to Radioactive Iodine (RAI) Therapy

The potential to resensitize tumors to RAI is one of the most significant therapeutic applications for phenolic compounds, though it’s important to note that this concept is currently a hypothesis based on in vitro evidence. The loss of NIS expression, which causes RAI resistance, is a key feature of dedifferentiated thyroid cancers [6,10]. As a compelling proof-of-concept in cell culture models, resveratrol has been shown to induce redifferentiation markers, including NIS, in anaplastic thyroid carcinoma cells by activating Notch1 signaling [54].

This in vitro success suggests a powerful therapeutic strategy: using a phenolic compound to “re-open the door” for iodine uptake, followed by RAI administration to destroy the newly sensitized cancer cells. However, this remains a preclinical hypothesis. Crucially, this redifferentiation effect has not yet been validated in in vivo animal models or clinical studies, representing a significant translational gap that must be bridged before this can be considered a viable strategy for patients [54].

**Figure 4:**
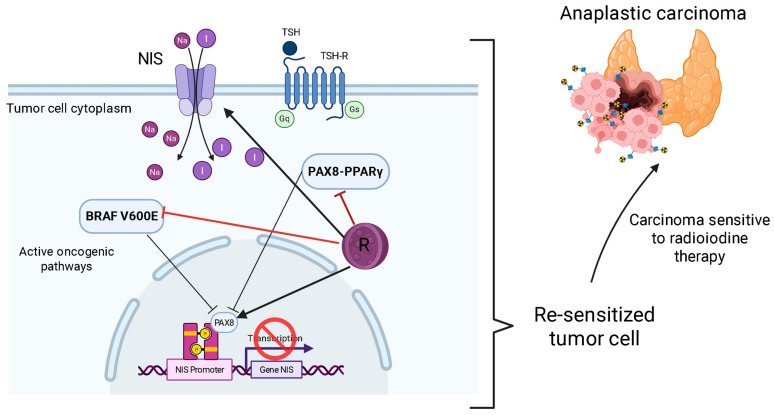
Molecular mechanisms underlying radioiodine sensitivity in anaplastic thyroid carcinoma (APC). The left panel illustrates the interaction of key signaling pathways within a tumor cell. The sodium-iodide symporter (NIS), responsible for iodide uptake, is shown in the cell membrane, transporting iodide (I-) along with sodium (Na+). The binding of thyroid-stimulating hormone (TSH) to its receptor (TSH-R) activates G proteins, which can influence downstream pathways. Oncogenic pathways, like those driven by the B-Raf proto-oncogene *V600E* (*BRAF V600E*) mutation, suppress the expression of the NIS, preventing iodine uptake. Thus, the resveratrol molecule blocks the activity of oncogenic pathways and directly acts on the NIS receptor gene expression. The right panel shows the clinical implication: an anaplastic carcinoma (above) can be resensitized to radioiodine therapy (below) through modulation of these molecular pathways, leading to a “resensitized tumor cell” capable of accumulating radioiodine and being sensitive to the therapy. This “redifferentiation” re-enables iodine uptake, potentially resensitizing the aggressive tumor to treatment with radioactive iodine (RAI). Created with BioRender.com (accessed on 1 August 2025).

Loss of NIS expression, primarily driven by activated MAPK and PI3K/Akt pathways, is a hallmark of dedifferentiation and a primary cause of RAI resistance in advanced thyroid cancers [6,10,64]. Phenolic compounds like resveratrol and curcumin show promise in reversing this process. They do so by inhibiting key oncogenic pathways and modulating the epigenetic state, for instance, through SIRT1 activation by resveratrol [6,42]. Resveratrol, for example, has been shown to induce redifferentiation markers (TTF1, TTF2, Pax8, NIS) in anaplastic thyroid carcinoma cells through the activation of Notch1 signaling [47] (Figure 4).

This redifferentiation creates a compelling possibility for a combination therapy: using phenolic compounds to “open the door” to the tumor, restoring iodine uptake, followed by the administration of RAI to selectively destroy the resensitized cancer cells [6,10]. This approach mirrors strategies observed with MAPK inhibitors, which successfully restored RAI avidity in previously refractory TCPD [10]. The ability to overcome RAI resistance, one of the most significant challenges in the treatment of advanced thyroid cancer, represents a significant advance that could transform the clinical management of these patients.

### 5.4. Enhancing with Checkpoint Immunotherapies

A more developed therapeutic angle, supported by compelling in vivo evidence, is the use of phenolic compounds to enhance the efficacy of ICIs. Advanced thyroid cancers often create an immunosuppressive microenvironment, rendering them “cold” and unresponsive to ICIs like anti-PD-1 [6]. Phenolic compounds can act as priming agents to “heat up” these tumors. This is not just a theoretical concept. An in vivo study using a murine model of anaplastic thyroid carcinoma demonstrated that curcumin enhances anti-tumor immunity by boosting CD8+ T cell function and downregulating the PD-L1 pathway. Furthermore, the study showed that combining curcumin with an anti-PD-1 antibody created a powerful synergistic effect, significantly improving tumor control [37]. While these animal model results are highly encouraging and provide a strong rationale for clinical trials, it’s essential to remember that in vivo success in mice does not always guarantee efficacy in humans. Clinical validation remains the necessary next step.

**Figure 5:**
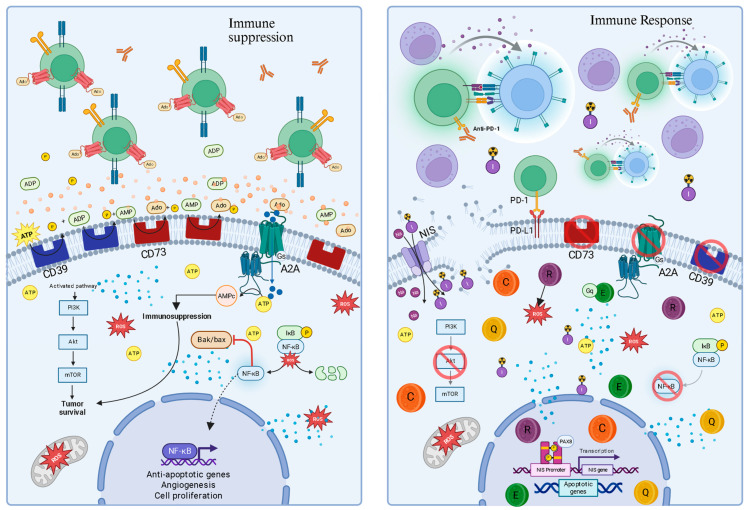
Differential effects of extracellular adenosine metabolism on immune suppression and immune response in the tumor microenvironment. The left panel illustrates a state of Immune Suppression. Extracellular ATP (eATP) is hydrolyzed by CD39 to ADP and subsequently by CD73 to adenosine (Ado). Adenosine binds to the A2A adenosine receptor (A2AR) on immune cells, activating downstream signaling pathways such as PKA and MAPK. This activation leads to the suppression of anti-tumor immune responses, promoting tumor survival through mechanisms like activation of Akt/mTOR and the transcription of anti-apoptotic genes, pro-angiogenic factors, and genes involved in cell proliferation via NF-κB. Reactive oxygen species (ROS) may also contribute to this suppressive environment. The right panel illustrates the therapeutic goal: using agents like phenolic compounds to inhibit CD73 and/or immune checkpoint inhibitors (ICIs) like anti-programmed cell death protein 1 (PD-1) antibodies to block the PD-1/programmed death-ligand 1 (PD-L1) axis. This combined intervention “heats up” the tumor, allowing immune cells to recognize and eliminate thyroid cancer cells. This shift promotes an active immune response, potentially leading to increased ROS production and the expression of pro-apoptotic genes (e.g., via altered *NIS* gene expression or other mechanisms, though the specific link to NIS requires further context from the broader study) and other molecules that favor tumor cell death and an effective anti-tumor immune response. Created with BioRender.com (accessed on 1 August 2025).

In this tumor “heating” scenario, anti-PD-1 therapies can then exert their full function, blocking the tumor’s second line of defense (the PD-1/PD-L1 interaction) and unleashing a robust anti-tumor immune response to eliminate cancer cells [6,68]. Specific examples, such as curcumin, have been shown to enhance anti-tumor immunity in CAT by elevating CD8+ T cell function and downregulating the AKT/mTORC1/STAT3/PD-L1 axis. Combined treatment with curcumin and anti-PD-1 further potentiates this anti-tumor immunity in vivo [37]. Recent preclinical work further exemplifies this synergy, using advanced biomimetic nanoparticles to co-deliver resveratrol and PD-L1 siRNA, achieving potent chemo-immunotherapy in endocrine cancer models [37,69]. Clinical trials for thyroid cancer involving ICIs, including combination therapies, are already underway, highlighting the clinical interest in enhancing immune responses [9]. This immune “priming” strategy, where phenolic compounds make the TME more permissive to immune attack, has profound clinical implications for expanding the patient population who benefit from ICIs in thyroid cancer (Figure 5).

### 5.5. Clinical Landscape of ICIs in Thyroid Cancer and the Rationale for Combination with Purinergic Modulators

While the principle of combining purinergic inhibitors with ICIs is supported by trials in other cancers, the specific landscape of ICI monotherapy in thyroid cancer provides the most direct rationale. Several key trials have established the activity of ICIs but also highlighted their limitations, particularly in less immunogenic subtypes. The table below summarizes pivotal trials of ICI monotherapy in advanced thyroid cancer, showing that while responses can be achieved—especially in aggressive subtypes like anaplastic thyroid cancer (ATC)—a substantial portion of patients do not benefit, indicating a strong presence of primary or acquired resistance.

The modest response rates to ICI monotherapy in both RAI-refractory DTC and ATC strongly suggest that simply “releasing the brakes” with anti-PD-1/PD-L1 is not enough for many patients [69,70]. This highlights a critical therapeutic gap that purinergic modulation is perfectly positioned to fill. The highly immunosuppressive, adenosine-rich TME, driven by overexpressed CD73, is a primary suspect in this resistance, as it can keep T cells exhausted even when the PD-1/PD-L1 pathway is blocked [22,31]. Therefore, a logical next step is to design clinical trials that directly test this hypothesis in thyroid cancer. A future study could be a Phase II randomized trial for patients with RAI-refractory DTC who have progressed on standard TKI therapy. The trial design could feature two groups (Arm A: Pembrolizumab (standard ICI) + Placebo; Arm B: Pembrolizumab + a CD73 inhibitor (e.g., Oleclumab) or a dual A2A/A2B antagonist).

The primary endpoint would be to determine if the combination significantly increases the Objective Response Rate (ORR) compared to ICI monotherapy. Key secondary endpoints should include analyzing tumor biopsies to confirm that the combination leads to a more favorable immune microenvironment (e.g., increased CD8+ T-cell infiltration and decreased T-cell exhaustion markers). Such a trial would directly test whether dismantling the “adenosine shield” can unlock the full potential of immunotherapy for patients with advanced thyroid cancer (Table 3).

### 5.6. Overcoming the Translational Challenge with Nanotechnology

A significant obstacle preventing the widespread clinical use of phenolic compounds is their inherently low systemic bioavailability, rapid metabolism, and poor water solubility [6,67,68]. The development of advanced nanodelivery systems, such as lipid or polymeric nanoparticles, is crucial to overcoming these pharmacokinetic limitations [6,69,70]. Nanoencapsulation can protect these compounds from premature degradation, significantly increase their solubility, and enable targeted delivery to tumor tissue through passive accumulation (e.g., enhanced permeability and retention (EPR) effect) or active targeting (e.g., ligand functionalization) [6,69,70]. This process ensures that the compounds reach effective therapeutic concentrations at the tumor site. For example, quantitative studies have shown that encapsulating curcumin in polymeric nanoparticles can increase its oral bioavailability by more than 15-fold compared to free curcumin, dramatically increasing plasma concentrations and extending its therapeutic window [74]. Similarly, the plasma half-life of resveratrol was extended from minutes to over 6 h using lipid-based nanocarriers, leading to greater tumor accumulation through the EPR effect [75].

The value of this approach has been clearly demonstrated in vivo in thyroid cancer models. Although free resveratrol failed to inhibit tumor growth in an animal model of anaplastic carcinoma, a sustained-release nanoparticle formulation (~200 nm in size) achieved significant tumor growth inhibition [76], a feat directly attributed to improved pharmacokinetics and sustained drug release at the tumor site. This vividly illustrates how nanotechnology can successfully translate a laboratory hypothesis into a validated in vivo result.

In this scenario, sustained and targeted resveratrol nanoparticles demonstrate effective inhibition of APC growth in vivo, a feat in which free resveratrol failed due to low bioavailability [66]. Similarly, nanoformulations of curcumin and quercetin have demonstrated enhanced anticancer activity due to improved bioavailability and targeted delivery [65,66], with recent reviews summarizing the latest nanotechnological advances for quercetin delivery in cancer treatment [76]. Nanotechnology is not just an enhancement but a key technology for the clinical translation of these compounds, essential for fully exploiting their therapeutic potential and benefiting patients with aggressive thyroid cancer.

The evolution of nanotechnology in this field is now moving toward multifunctional “smart” platforms. Researchers are developing advanced nanoparticles capable of co-delivering phenolic compounds and immune checkpoint inhibitors to the tumor site simultaneously [16,17,77]. The rationale is to use the phenolic compound to remodel the immunosuppressive tumor microenvironment—for example, by inhibiting CD73 and reducing adenosine—thus sensitizing the tumor to a more potent attack by the co-administered ICI. These bioinspired nanomedicines represent the next therapeutic frontier, aiming to create a synergistic effect far more powerful than administering either agent alone [77,78,79] (Table 4).

### 5.7. Limitations of Current Evidence

Despite the exciting potential of phenolic compounds, their path to clinical application is hindered by several major, unresolved challenges.

#### 5.7.1. The Overwhelming Bioavailability Hurdle

This is the central obstacle. Nearly all compelling findings for phenolics in thyroid cancer come from preclinical studies using concentrations that are not achievable in humans through diet or unformulated supplements [41,42,44].

#### 5.7.2. Pleiotropy as a Double-Edged Sword

The ability of phenolics to hit multiple targets is often cited as a strength [6], but it also implies a lack of specificity that can lead to unpredictable off-target effects and false bioassay results [53].

### 5.8. Discussion on Clinical Trials and Research Gaps

Despite compelling preclinical evidence and mechanistic insights, the translation of phenolic compounds into established clinical therapies for thyroid cancer remains a challenge. Human intervention studies for phenolics generally show less clear results compared to in vitro or animal models, often suggesting that concentrations higher than those achievable through diet may be required for therapeutic effects [11,12,64,68].

There is a critical need for more dedicated clinical trials to rigorously assess the safety, optimal therapeutic dosages, and precise mechanisms of action of phenolic compounds, both as monotherapies and in combination with conventional or immunotherapeutic agents, specifically in thyroid cancer patients [51,65,69]. Furthermore, the impact of immunosenescence on immunotherapy efficacy, particularly in elderly cancer patients, represents a significant challenge that needs to be addressed in future clinical trial designs [6,65]. Conducting these trials is the next crucial step to bridge the gap between promising laboratory discoveries and tangible patient benefits (Table 5).

## 6. Conclusions and Future Perspectives

The synergistic interaction between oxidative stress, chronic inflammation, and purinergic signaling constitutes a central pathophysiological axis in the development, progression, and immune evasion of thyroid cancer. The gland’s intrinsic metabolic vulnerability to oxidative stress establishes a microenvironment conducive to carcinogenesis, in which the purinergic system acts as a molecular translator. This system converts cellular damage signals, such as extracellular ATP, into an immunosuppressive and pro-growth microenvironment, orchestrated by the CD39/CD73 ectonucleotidase duo and the dual biology of the P2X7 receptor. In this context, the view of phenolic compounds as mere antioxidants is reductive; accumulating evidence positions them as sophisticated immunometabolic modulators with the ability to reprogram this axis. By simultaneously targeting the adenosine pathway and intracellular survival pathways, these phytochemicals offer a multi-targeted strategy to reverse immunosuppression and induce tumor death. The translational potential of this approach is multifaceted. The ability of these compounds to act as immunotherapeutic primers, converting immunologically “cold” tumors into “hot” ones by dismantling the adenosine shield, represents a promising avenue for expanding the efficacy of immune checkpoint inhibitors in thyroid cancer. Additionally, their ability to induce cellular redifferentiation and restore sodium-iodide symporter (NIS) expression offers an innovative strategy for potentially resensitizing tumors refractory to radioactive iodine therapy, overcoming one of the field’s most significant clinical challenges.

However, translating these concepts into clinical practice faces significant challenges that shape future research directions. First, advancing precision oncology requires the development of robust biomarkers. The identification of predictive markers—such as CD39, CD73, and A2AR expression levels, or adenosinergic gene signatures in the TME—will be crucial for selecting patients with a higher likelihood of response. Second, the remarkable heterogeneity of thyroid cancer requires in-depth investigation of purinergic components in different histological and molecular subtypes (e.g., BRAF vs. RAS mutations). Third, it is imperative to anticipate and overcome resistance mechanisms, such as adenosine production through alternative pathways (via CD38), which may require double or triple blockade strategies. Finally, the main obstacle to the clinical application of phenolic compounds lies in their pharmacokinetic limitations. The development of effective nanoformulations is an indispensable step to overcome low bioavailability and ensure targeted tumor delivery, which is the greatest translational challenge to be overcome. Future research will undoubtedly focus on next-generation “smart” drug delivery systems capable of responding to the tumor microenvironment and co-delivering multiple therapeutic agents for a synergistic effect.

Therefore, the next steps in research should focus on rigorous preclinical validation of these hypotheses. It is crucial to explore combinations of purinergic modulators, including nanostructured phenolic compounds, with standard targeted therapies for TC, such as BRAF/MEK inhibitors. The scarcity of direct data on purinergic modulation in thyroid cancer highlights the critical need for investigations in relevant preclinical models—such as cell lines, patient-derived organoids, and animal models—to accelerate the translation of these promising concepts and ultimately offer new therapeutic strategies for patients with the most aggressive forms of the disease.

## Figures and Tables

**Table 1:** Main Modulating Agents of Purinergic Signaling in Clinical Trials for Solid Tumors.

Compound (Name/Code)	Molecular Target	Mechanism of Action	Highest Clinical Phase	Cancer Types
TTX-030	CD39	Allosteric inhibitory antibody	Phase I	Solid tumors (NCT04318536)
IPH5201	CD39	Inhibitory antibody	Phase I	Solid tumors (NCT04318536)
Oleclumab (MEDI9447)	CD73	Non-competitive inhibitory antibody	Phase III (initiated)	Lung cancer, pancreatic, colorectal (NCT02503923)
CPI-006	CD73	Competitive inhibitory antibody	Phase I/II	Solid tumors, lymphoma (NCT03454451)
Etrumadenant (AB928)	A2AR/A2BR	Small molecule dual antagonist	Phase II	Solid tumors (colorectal, prostate) (NCT03719129, NCT03719131)
Ciforadenant (CPI-444)	A2AR	Small molecule antagonist	Phase I/II	Renal cancer (NCT02657889) and solid tumors (NCT03167169)

**Table 2:** Mechanisms of Action of Phenolic Compounds in Thyroid Cancer.

Phenolic Compound	Key Molecular Targets	Primary Effects on Cancer Hallmarks	Specific Modulation of Oxidative Stress	Specific Modulation of Inflammation	Specific Modulation of Purinergic Signaling	Reference
Curcumin	PI3K/Akt/mTOR, NF-κB, STAT3, TGF-β/Smad2/3, MAPK, miR-301a-3p	Inhibits proliferation, induces apoptosis, inhibits migration/invasion, inhibits metastasis, and overcomes drug resistance	Prooxidant effect (ROS generation) causes mitochondrial damage	Inhibits NF-κB, modulates inflammatory cytokines	Decreases expression of CD39, CD73, and A2A	[37,38,39,40,43,44,45]
Resveratrol	SIRT1, Adenosine Receptors (A1, A2A), Notch1, Caspases	Induces redifferentiation, inhibits cell growth, induces cell death, reverses multidrug resistance, sensitizes to ^131^I	Induces cell death via oxidative stress, protects against ROS damage	Anti-inflammatory	Increases A1, reduces A2A	[47,48,49,50,51]
Quercetin	CD73, NAG-1/GDF15, PI3K/Akt/mTOR, MAPK/ERK, NF-κB, JAK/STAT, MMP3	Induces apoptosis, inhibits proliferation, inhibits angiogenesis, inhibits migration/invasion, causes cell cycle arrest	Potent antioxidant (neutralizes ROS), mitigates oxidative stress	Anti-inflammatory, downregulates NF-κB, modulates JAK/STAT	Inhibits CD73 activity, a potential limitation of ATP efflux	[42,54,55,56,57,58]
EGCG	CD73, Bcl-2, EGFR/RAS/RAF/MEK/ERK, NF-κB, MAPK, AMPK, DNA methyltransferase	Modulates apoptosis, inhibits growth/proliferation, inhibits migration/invasion, inhibits angiogenesis, causes cell cycle arrest, inhibits EMT	Dual function (antioxidant and prooxidant), ROS generation	Anti-inflammatory, inhibits NF-κB	Inhibits CD73	[36,59,60,61,62]

**Table 3:** Summary of Key Clinical Trials of Immune Checkpoint Inhibitors (ICIs) in Thyroid Cancer.

Trial Name/Identifier	Drug(s)	Phase	Thyroid Cancer Subtype	Key Outcome (ORR)	Reference
KEYNOTE-158 (NCT02628067)	Pembrolizumab (Anti-PD-1)	II	RAI-Refractory DTC	9.1%	[70]
LENVIMA/KEYTRUDA (NCT02973997)	Pembrolizumab + Lenvatinib (TKI)	II	RAI-Refractory DTC	64.3%	[71]
SELECT (NCT01321554)	Lenvatinib (TKI)	III	RAI-Refractory DTC	64.8%	[72]
NCT03246958	Nivolumab (Anti-PD-1) + Ipilimumab (Anti-CTLA-4)	II	RAI-Refractory DTC	9.4%	[73]
LENVIMA/KEYTRUDA (NCT0297397)	Pembrolizumab + Lenvatinib (TKI)	II	RAI-Refractory DTC	64.3%	[70]

**Table 4:** Examples of Nanocarrier Systems for Phenolic Compound Delivery in Cancer Models.

Phenolic Compound	Nanocarrier Type	Particle Size (nm)	Drug Loading/Encapsulation Efficiency	Study Model	In Vivo Efficacy Highlights	Reference
Curcumin	Solid Lipid Nanoparticles (SLNs)	~40 nm	23.38% Loading/72.47% Encapsulation	Breast Cancer (SKBR3 cells, in vitro)	Showed higher cytotoxicity and apoptosis induction in SKBR3 cells compared to free curcumin.	[77]
Resveratrol	Targeted Polymeric Nanoparticles (IL-13Rα2-targeting)	~30 nm	6.81% Loading/40.84% Encapsulation	Anaplastic Thyroid Cancer (Xenograft Mice)	Inhibited tumor growth by 69.23% compared to the untreated group (*p* < 0.01), without the toxicity of chemotherapy.	[78]
Quercetin	Folic Acid-EPA-Liposomes	106.4 nm	92.69% Encapsulation	Cervical (HeLa) and Liver (HepG2) Cancer cells (in vitro)	16-fold increase in potency vs. free quercetin in HeLa cells (IC50: 3.76 µg/mL); showed low toxicity in healthy cells.	[79]
EGCG	Liposomes	~100 nm	>80% Encapsulation	Prostate Cancer (Xenograft Mice)	Increased tumor accumulation by 4-fold and significantly improved tumor suppression vs. free EGCG.	[80]

**Table 5:** Relevant Preclinical and Clinical Trials of Phenolic Compounds or Related Therapies in Thyroid Cancer.

Compound/Therapeutic Strategy	Study Type	Thyroid Cancer Model/Context	Main Finding/Outcome
Curcumin	In vitro	PTC cells (BCPAP, TPC-1)	Inhibits proliferation, migration, and invasion; induces apoptosis and autophagy [38,39,40]
	In vivo (murine model)	APC xenografts	Enhances CD8+ T cell function and downregulates AKT/mTORC1/STAT3/PD-L1 axis; synergistic with anti-PD-1 [37]
	Preclinical	PTC cells	Inhibits metastasis via downregulation of the TGF-β/Smad2/3 pathway [43,44,45]
Resveratrol	In vitro	APC cells (HTh7, 8505C)	Induces expression of differentiation markers (NIS) via Notch1 activation; suppresses cell growth [47,48,49,50,51]
	In vitro	Thyroid cancer cells	Increases ^131^I-induced cell death; protects normal cells from ^131^I toxicity [46,47,48,49,50]
	In vivo (nanoparticles)	APC xenografts	Sustained-release nanoparticles effectively inhibit tumor growth in vivo, overcoming the poor bioavailability of free resveratrol [66]
Quercetin	In vitro	Differentiated thyroid cancer cells	Induces anticancer activity via upregulation of pro-NAG-1/GDF15; induces apoptosis and cell cycle arrest [54,55,56,57,58]
	In vitro	Thyroid cancer cells	Inhibits proliferation and MMP3 expression under high glucose conditions [2]
	Preclinical	PTC cells	Demonstrates strong anti-tumor efficacy; regulates TNF, PI3K-AKT, NF-κB pathways [61,62]
EGCG	In vitro	Human thyroid carcinoma cells	Inhibits growth and enhances apoptosis via suppression of EGFR/RAS/RAF/MEK/ERK signaling pathway [59]
	In vitro	Human thyroid carcinoma cells	Decreases migration and invasion; inhibits proliferation and motility with loss of EMT markers [59,60,61,62]
Nanoformulations	General	Various cancer types, including thyroid	Improve bioavailability, solubility, and stability; enable targeted tumor delivery [6,36,62,65]
ICIs (anti-PD-1/PD-L1)	Clinical	Thyroid cancer	Ongoing trials, including monotherapy and combinations; investigation of biomarkers [9]

## Abbreviations

The following abbreviations are used in this manuscript:

TC	Thyroid Cancer
TME	Tumor Microenvironment
BRAF	proto-oncogene B-Raf
RAS	rat sarcoma virus
p53	tumor suppressor protein or TP53
EGCG	Epigallocatechin-3-gallate
ATP	Adenosine Triphosphate
Ado	Adenosine
NF-κB	Nuclear Factor kappa B
OS	Oxidative Stress
PTC	Papillary Carcinoma
DTC	Differentiated Thyroid Carcinomas
FTC	Follicular Carcinoma
APC	Anaplastic Carcinoma
DAMP	Damage-Associated Molecular Pattern
MAPK	MAP Kinase
RET	Rearranged during transfection
PPAR	Peroxisome Proliferator-Activated Receptor
PPFP	PAX8/PPARY fusion protein
NIS	Sodium/Iodine Symporter
RAI	Radioactive Iodine
TKIs	Tyrosine Kinase Inhibitors
H_2_O_2_	Hydrogen Peroxide
TPO	Thyroid Peroxidase
GSH	Glutathione
TAS	Total Antioxidant Status
TOS	Total Oxidant Status
OSI	Oxidative Stress Index
NOX4	NADPH Oxidase 4
DUOX	Dual Oxidase
8-OHdG	8-hydroxy-2′-deoxyguanosine
PI3K	Phosphoinositol-3-kinase
AKT	Protein Kinase B
PTPs	Protein Tyrosine Phosphatases
ERα	Estrogen Receptor Alpha
ERK1/2	Extracellular signal-regulated kinases 1 and 2
CT	Thyroid Cancer
IL-6	Interleukin-6
CXCL8	Chemokine 8
eATP	Extracellular ATP
P1	Purinoreceptors type 1
P2	Purinoreceptors type 2
P2X7	Purinoreceptor P2X subtype 7
CD39	Ectonucleoside Triphosphate Diphosphohydrolase E-NTPDase1
CD73	Ecto-5′-nucleotidase
AMP	Adenosine Monophosphate
TGF-β	Transforming Growth Factor beta
NK	Natural Killer (Natural killer cells)
cAMP	Cyclic AMP (3′-5′-cyclic adenosine monophosphate)
Tregs	Regulatory T Cells (regulatory T lymphocytes)
PD-1	Programmed Cell Death Protein 1
PD-L1	Programmed Cell Death Ligand 1
nfP2X7	Non-functional P2X7 receptor
NLRP3	Inflammasome coding gene
IL-1β	Interleukin-1 beta
IL-18	Interleukin type 18
NADPH	Nicotinamide Adenine Dinucleotide Phosphate Reduced
OXPHOS	Mitochondrial Oxidative Phosphorylation
ABCB6	ATP-Binding Cassette Transporter Subfamily B, Member 6
PANX-1	Pannexin-1
P2X	Ionotropic P2 purinoreceptors
P2Y	Metabotropic P2 purinoreceptors
ADA	Adenosine Deaminase
ICIs	Immune Checkpoint Inhibitors
PKA	Protein Kinase A
Akt/mTOR	protein kinase B/mammalian target of rapamycin
NAG-1/GDF15	Non-Steroidal Anti-Inflammatory Drug-Activated Gene-1/Growth Differentiation Factor 15
MMP3	Matrix Metalloproteinase-3
JAK/STAT	Janus kinase/signal transducer and activator of transcription
EGFR	Epidermal Growth Factor Receptor
SIRT1	Sirtuin 1
EMT	Epithelial-Mesenchymal Transition
EPR	Enhanced Permeability and Retention
CD38	Differentiation cluster 38
